# The Association of Hydration Status with Physical Signs, Symptoms and Survival in Advanced Cancer—The Use of Bioelectrical Impedance Vector Analysis (BIVA) Technology to Evaluate Fluid Volume in Palliative Care: An Observational Study

**DOI:** 10.1371/journal.pone.0163114

**Published:** 2016-09-27

**Authors:** Amara Callistus Nwosu, Catriona R. Mayland, Stephen Mason, Trevor F. Cox, Andrea Varro, John Ellershaw

**Affiliations:** 1 Marie Curie Palliative Care Institute Liverpool (MCPCIL), University of Liverpool, Liverpool, United Kingdom; 2 Liverpool Cancer Trials Unit, University of Liverpool, Liverpool, United Kingdom; 3 School of Physiological Sciences, University of Liverpool, Liverpool, United Kingdom; Taipei Medical University, TAIWAN

## Abstract

**Background:**

Hydration in advanced cancer is a controversial area; however, current hydration assessments methods are poorly developed. Bioelectrical impedance vector analysis (BIVA) is an accurate hydration tool; however its application in advanced cancer has not been explored. This study used BIVA to evaluate hydration status in advanced cancer to examine the association of fluid status with symptoms, physical signs, renal biochemical measures and survival.

**Materials and methods:**

An observational study of 90 adults with advanced cancer receiving care in a UK specialist palliative care inpatient unit was conducted. Hydration status was assessed using BIVA in addition to assessments of symptoms, physical signs, performance status, renal biochemical measures, oral fluid intake and medications. The association of clinical variables with hydration was evaluated using regression analysis. A survival analysis was conducted to examine the influence of hydration status and renal failure.

**Results:**

The hydration status of participants was normal in 43 (47.8%), 'more hydrated' in 37 (41.1%) and 'less hydrated' in 10 (11.1%). Lower hydration was associated with increased symptom intensity (Beta = -0.29, p = 0.04) and higher scores for physical signs associated with dehydration (Beta = 10.94, p = 0.02). Higher hydration was associated with oedema (Beta = 2.55, p<0.001). Median survival was statistically significantly shorter in 'less hydrated' patients (44 vs. 68 days; p = 0.049) and in pre-renal failure (44 vs. 100 days; p = 0.003).

**Conclusions:**

In advanced cancer, hydration status was associated with clinical signs and symptoms. Hydration status and pre-renal failure were independent predictors of survival. Further studies can establish the utility of BIVA as a standardised hydration assessment tool and explore its potential research application, in order to inform the clinical management of fluid balance in patients with advanced cancer.

## Introduction

People with advanced cancer commonly experience reduced oral intake in the last days of life.[1] This may cause healthcare professionals and family caregivers to question whether clinically assisted hydration (CAH) is required for the management of hydration status and symptoms. However, there is limited evidence to determine the association between hydration and symptoms in advanced cancer.[2] Physical examination has low sensitivity and specificity for identifying fluid deficit.[2, 3] The evidence for the use and effects of CAH in advanced cancer is limited, conflicting and inconclusive.[2, 4, 5]

Bioelectrical impedance analysis (BIA) is a non-invasive body composition assessment tool based on the flow of electrical current through the body.[6] The recorded measurements include: resistance (R—the restriction to the flow of electrical current through the body, primarily related to the amount of water present in tissue) and reactance (Xc—resistive effect produced by the tissue interfaces and cell membranes). BIA technology has been used to evaluate hydration and nutrition in various populations.[2, 7] The impedance index (Height—H (m)^2^/R (Ohms)) is the best single predictor of total body water (TBW) in validation studies, including cancer populations.[8–19]

The BIA vector analysis (BIVA) RXc graph method involves BIA measurements that are standardized by height and plotted as bivariate vectors with their confidence intervals (which are ellipses on the R-Xc plane). The advantage of this method is that it allows for information to be obtained simultaneously about changes in tissue hydration or soft-tissue mass, independent of regression equations, or body weight. BIVA has been used to study hydration in a variety of different diseases[20–28] and to undertake general body composition assessments in lung cancer[27, 29] and cancers of the head and neck.[30]

### Aim

The aim of this observational study was to use H^2^/R and BIVA to study the hydration status of individuals with advanced cancer, in order to determine the relationship of hydration with symptoms, physical signs, renal biochemical measures and survival.

## Materials and Methods

Participants were recruited from a UK specialist palliative care unit between December 2012 and October 2013. The research project adhered to the requirements of the UK Department of Health Research Governance Framework. Written consent was obtained from all study participants; this included consent to report individual patient data in publication. Participant consent was recorded in a research recruitment log. This study was approved by the North Wales Research Ethics Committee–West (Local research ethics committee approval number = 12/WA/0200). The eligibility criteria for study entry was: admission to specialist palliative care inpatient unit; age ≥18 years; cancer (proven by histology or radiological imaging); palliative condition (no further curative treatment possible); able to understand and communicate in English; serum urea and creatinine recorded by the clinical team in the previous 72 hours. Our exclusion criteria were: individuals with implantable defibrillator devices; unable to provide fully informed consent; active transmissible infections; current use of CAH; current antineoplastic treatment.

### Assessments

All assessments were conducted between 9am–12pm. The following information was recorded: age (years); gender; ethnicity; cancer diagnosis (defined by the International Classification of Diseases)[31] and primary site of cancer.

#### Participant observations

A dehydration score was calculated using the approach of Morita et al,[32] based on a total of scores from three physical findings: oral mucous membranes moisture (0: moist, 1: somewhat dry, 2: dry), axillary moisture (0: moist, 1: dry), and sunkenness of eyes (0: normal, 1: slightly sunken, 2: sunken). These signs have significant correlations with biological dehydration,[33–36] with higher scores (range 0–5) indicating an increased risk of dehydration (previous studies have used a Morita cut-off of 2 to define an increased risk of dehydration[37, 38]). Performance status was recorded using the Eastern Cooperative Oncology Group (ECOG) scale (0 = fully active, 5 = dead).[39] Daily fluid intake (0–199mL, 200–499mL, 500–799 or >800mLs) was recorded using nursing assessments. Height was measured, without shoes, to the nearest 0.1cm using a portable stadiometer (SECA 213 Height Measure / Stadiometer). Length was measured in those unable to stand. Body weight was measured to the nearest 0.1kg (SECA 955 High Capacity Electronic Chair Scale). The following biochemical measures were recorded: urea (mmol/L), creatinine (μmol/L), estimated glomerular filtration rate (eGFR) (mL/min/1.73m^2^), serum sodium (mmol/L), serum albumin (g/L), adjusted calcium (mmol/L) and urine osmolality (mosm/kg).

#### Hydration questionnaire

Participants completed a hydration symptom questionnaire (Burge-4 score).[36, 40] This comprised of four questions (thirst, dry mouth, unpleasant taste and fatigue) measuring symptom severity over the previous 24-hours using a 100mm Visual Analogue Scale (VAS). At the time of the study, the Burge questionnaire was the only available dehydration symptom assessment tool for advanced cancer patients.

#### Medication review

The following medication information was recorded: the total daily morphine dose (calculated using opioid-equivalency ratios[41, 42]); use of serotonin reuptake inhibitors (SSRIs); use of serotonin noradrenaline reuptake inhibitors (SNRIs) and diuretics. The Anticholinergic Burden (ACB) scale[43, 44] was used to calculate the potential anticholinergic symptom burden from the use of these medications.

#### Bioelectrical impedance assessments

The EFG^3^ ElectroFluidGraph Vector Impedance Analyser (Akern) was used for the BIA assessments. The method involved a tetra-polar technique to deliver a single frequency electrical current of 50kHz (±5%). The external calibration of the analyser was checked daily using an impedance calibration circuit (R = 470 Ω, Xc = 90 Ω). The testing procedure was conducted in line with methods described by Lukaski[45] and other recommendations.[46, 47] Participants were lightly clothed, lying in the supine horizontal position, without shoes or socks. Their arms were positioned 30 degrees from the body with the legs positioned 45 degrees away from each other. Two disposable pre-gelled aluminium electrodes were affixed to the dorsum of the right hand (one placed on the edge of an imaginary line bisecting the ulnar head and the other on the middle finger proximal to the metacarpal-phalangeal) and two to the dorsum of the right foot (one placed medially, to an imaginary line bisecting the medical malleolus at the ankle and the other proximal to the metatarsal-phalangeal joints).

#### BIVA point graph analysis

For the BIVA method, the impedance vector (Z) was plotted as a bivariate vector from its components, R (X axis) and Xc (Y axis), after being standardized by height (H); this forms two correlated normal random variables (i.e. a bivariate Gaussian vector).[48, 49] Elliptical probability regions of the mean vector are plotted on the RXc plane forming elliptical probability regions on the RXc plane, which are tolerance ellipses for individual vectors and confidence ellipses for mean vectors.[6, 49–52] Tolerance ellipses are the bivariate reference intervals of a normal population for an observation. The RXc graph features three tolerance ellipses: the median, the third quartile, and the 95th percentile (i.e. 50%, 75% and 95% of individual points).

**Fig 1:**
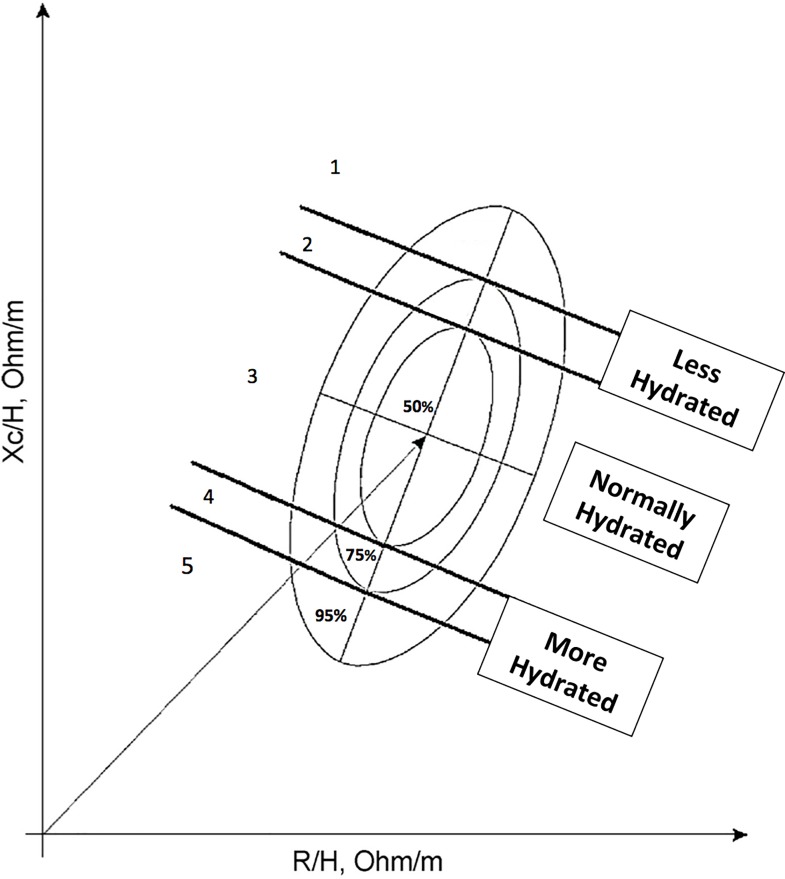
Classification of hydration status using the RXc graph and the 50^th^ and 75^th^ percentile tolerance ellipses. The 50% and 75% percentiles were used to project a 5-point hydration scale on the BIA normogram. Positions 1 and 2 = ‘less hydrated’ individuals; position 3 = ‘normally hydrated’ individuals; positions 4 and 5 = ‘more hydrated’ individuals.

Participant data were plotted on the RXc point graph using the 50%, 75% and 95% tolerance ellipses from a non-cancer reference population.[6] Hydration status was determined by the individual’s baseline bioimpedance vector position on the BIVA RXc normogram. The normogram is a five-point graph (corresponding with the boundaries of each tolerance ellipse). We simplified the normogram into three parallel sections for this study (Fig 1). Individuals with vectors falling in (or above) the 51–75% tolerance ellipse (points 1 and 2) were classified as ‘less-hydrated’. Participants with vectors in the central 50^th^ percentile ellipse (point 3) were classified as ‘normally-hydrated’. Those with vectors in (or below) the lower 51%–75% percentile range (points 4 and 5) were classified as ‘more hydrated’. Participants were compared according to their hydration status classification (‘less hydrated’ vs. ‘not less hydrated’) to evaluate differences in biochemistry, symptoms, clinical signs and fluid intake.

#### H^2^/R Analysis

The H^2^/R was used as a proxy measure of hydration status (i.e. TBW). Multiple linear regression analysis was conducted to further study the relationship between several predictor variables with the H^2^/R. The variables included: patient demographics (age, gender), clinical measurements (Morita Dehydration Score, oedema presence) serum biochemistry (urea:creatinine (ur:cr) ratio) and self-reported symptoms (Burge-4 score). Pre-renal failure was defined by a ur:cr ratio of ≥100 (mmol/mmol). This biochemical definition was chosen based on the work of similar studies (NOTE: as creatinine was recorded in μmol/L we divided this by 1000 to convert to mmol/L to calculate the ur:cr ratio).[3, 53–55] A separate multiple linear regression analysis involving the Burge-4 score and the assessed medications was conducted to determine the potential influence of medications on symptoms.

### Sample size calculation

An exploratory sample of 90 patients was chosen to achieve a minimum of 10 subjects for each item in the regression model. For the RXc graph, a sample size of 90 provides a 95% confidence region for the mean vector of the vector random variable (Z(R), Z(Xc)) as an ellipse with semi-axes of approximate lengths of 0.33 and 0.16. For the two-group analysis, a sample size of 45 (for each of the two groups) has power of 0.8 for detecting a difference of (0.5, 0.5) in the mean BIVA vectors between groups, for significance level of 0.05.[56]

### Statistical analysis

The primary focus of this study was to use the H^2^/R and BIVA to evaluate the relationship of hydration status with physical signs, symptoms, biochemical measures and survival. Statistical Package for the Social Sciences (SPSS) version 21.0 was used for standard calculations. Distributions of all variables were assessed for normality using the Shapiro-Wilk test. Parametric and non-parametric tests were used as appropriate. Frequency analysis was conducted to compare differences between groups and variables using the chi-squared test, Student t test and the Mann-Whitney U test. For the independent t-tests, Levene’s test for homogeneity of variance was used to examine the quality of variances within a population to identify whether derivatives required exclusion or separate analysis from the cohort. Multiple linear regression analysis was used to evaluate associations between variables. The significance level was set at <0.05. The BIVA statistical analysis was conducted using software developed by Professor Antonio Piccoli, University of Padova.[57] Hotelling’s T^2^ test for vector analysis was used to compare for significant difference between mean vector distances.

Survival was evaluated from baseline assessment to death. All patients were followed up for 3-months following completion of the study. Kaplan-Meier analysis was used to analyse survival, according to the hydration status and renal failure status. A Cox proportional hazards model was used to assess the effect of the ur:cr ratio and the H^2^/R on survival, with adjustment for demographic characteristics, baseline ECOG performance status and cancer type.

## Results

### Demographics

**Fig 2:**
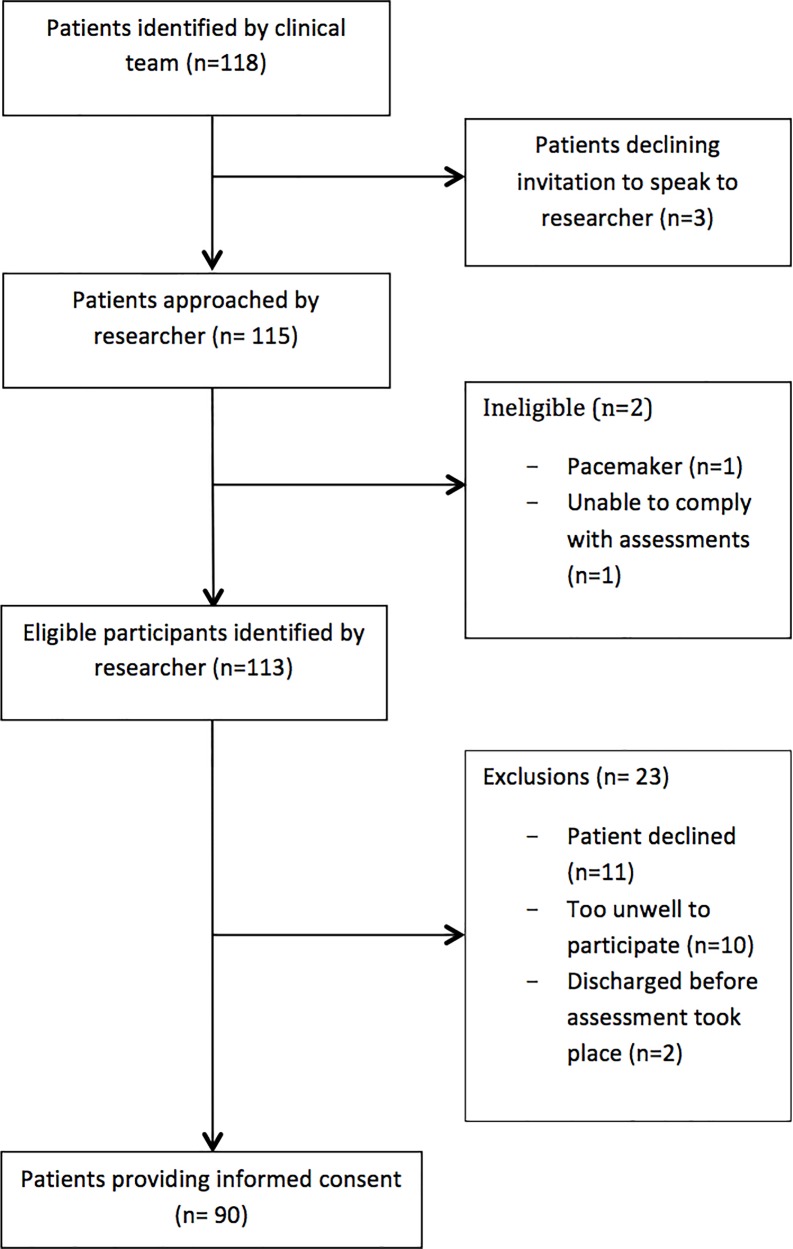
Recruitment flowchart. Flowchart representation of the number of individuals recruited to the study.

Ninety patients (males = 42, 46.7%; females = 48, 53.3%) participated (recruitment rate = 76.3%) (Fig 2). The mean age of participants was 71.2 years (SD± 12.21) and were mostly Caucasian (n = 89, 98.9%) (Table 1). Twenty-one different types of cancers (in addition to 4 (4.4%) unknowns) were recorded; lung cancer was the most common (n = 14, 15.6%). Most participants had an ECOG performance status of 3 (n = 36, 40%).

**Table 1:** Demographic details of study participants.

Characteristic	N (Data presented as mean or %)
Mean age (± SD), years	71.17 (12.21)
Male	42 (46.7)
Female	48 (53.3)
Mean height (± SD), cm	164.22 (9.6)
Mean weight (± SD), kg	69.45 (17.9)
Mean body mass index (± SD), kg/m^2^	25.17 (4.98)
*Race/ethnicity*	
Caucasian	89 (98.9)
Other	1 (1.1)
*ECOG*	
0: Asymptomatic	0 (0)
1: Symptomatic but completely ambulatory	15 (16.7)
2: Symptomatic, <50% in bed during the day	22 (24.4)
3: Symptomatic, >50% in bed, but not bedbound	36 (40.0)
4: Bedbound	17 (18.9)
*Cancer diagnosis*	
Lung	14 (15.6)
Colorectal	11 (12.2)
Prostate	10 (11.1)
Ovarian	6 (6.7)
Breast	6 (6.7)
Oesophageal	5 (5.6)
Myeloma	5 (5.6)
Pancreatic	4 (4.4)
Unknown	4 (4.4)
Cervical	3 (3.3)
Mesothelioma	3 (3.3)
Gastric	3 (3.3)
Brain	3 (3.3)
Melanoma	2 (2.2)
Soft tissue/muscle/connective tissue	2 (2.2)
Biliary	2 (2.2)
Lymphoma	2 (2.2)
Bladder	1 (1.1)
Liver	1 (1.1)
Groin	1 (1.1)
Uterus	1 (1.1)
Tongue	1 (1.1)

Table 1 shows the details of age, gender, height, weight, body mass index, performance status and cancer diagnosis.

### Clinical assessments

The baseline assessments are presented in Table 2. Most participants had a daily oral fluid intake of approximately 500–799mLs (n = 42, 46.7%). Pre-renal failure was present in 37 (41.1%) patients and mean eGFR was 72.1 mL/min/1.73m^2^. The highest symptom score was recorded for fatigue (M = 63.60, SD = 30.09). The mean Burge-4 score was 222.07mm (SD = 95.40) and ranged from 0 to 400mm. The Morita Dehydration Score was ≥2 in 52 (57.8%) participants.

**Table 2:** Study baseline results.

Variable	N	Mean (M)	SD	Range (min–max)	Normal reference range (min–max)
*Biochemical results*					
Sodium (mmol/L)	89	136.12	4.28	126–145	133–146
Urea (mmol/L)	90	7.26	4.36	1.3–33.8	2.5–7.8
Creatinine (μmol/L)	90	79.26	30.33	23–183	50–130
eGFR (mL/min/1.73m^2^)	90	72.1	18.77	24–90	0–90
Ur:cr ratio (mmol/mmol)	90	96.68	53.16	32.61–383.61	
Pre-renal failure: Ur:cr ratio ≥100 (%)	37 (41.1)				
Adjusted calcium (mmol/L)	89	2.32	0.24	1.65–3.5	2.20–2.60
Serum albumin (g/L)	90	32.07	6.08	3–47	35–50
Serum osmolarity (mmol/kg)	61	286.36	10.03	260–311	275–295
Urine osmolarity (mmol/kg)	22	511.77	202.83	174–951	250–750
*Bioelectrical impedance*					
R/H (Ohm/m)	90	341.58	82.22		
Xc/H (Ohm/m)	90	27.68	9.49		
PA (degrees)	90	4.71	1.33		
H^2^/R (m^2^/Ohm)	90	51.58	15.41		
*Morita Dehydration Score*					
<2 (%)	38 (42.2)				
≥2 (%)	52 (57.8)				
*Burge-4 score (mm)*					
Total score (mm)	90	222.07	95.40	0–400	
Thirst (mm)	90	56.11	29.49	0–100	
Dry mouth (mm)	90	60.01	30.64	0–100	
Unpleasant taste (mm)	90	42.34	34.11	0–100	
Fatigue (mm)	90	63.60	30.09	0–100	
*Daily oral fluid intake*					
0–499mLs (%)	27 (30.0)				
500–799mLs (%)	42 (46.7)				
≥800mLs (%)	21 (23.3)				

Table 2 shows descriptive statistics of biochemical results, bioelectrical impedance, physical signs (Morita Dehydration Score), symptoms (Burge-4 score) and daily oral fluid intake.

### Multiple linear regression analysis

The H^2^/R was significantly predicted by female gender (Beta = -13.85, p<0.001), symptoms (the Burge-4 score) (Beta = -0.29, p = 0.04), physical signs (the Morita Dehydration Score) (Beta = -2.55, p = 0.02) and oedema (Beta = 10.94, p<0.001) (Table 3). A separate regression analysis demonstrated that opioids, diuretics, anticholinergics, SNRIs and SSRIs medications were not statistically significant in predicting symptoms (Burge-4 score).

**Table 3:** Multiple linear regression analysis of the impedance index (H^2^/R).

Variable	Beta (standard error)	P
Constant	96.96 (10.02)	<0.001
Age	0.13 (0.11)	0.246
Female	-13.85 (2.52)	<0.001
ECOG	-0.55 (1.38)	0.692
Oedema present	10.94 (2.89)	<0.001
Urea:creatinine ratio	-0.02 (0.02)	0.423
Morita Dehydration Score	-2.55 (1.1)	0.023
Burge-4 score	-0.29 (0.14)	0.038
R	0.71	
R squared	0.50	
Adjusted R squared	0.45	
Standard error of estimate	11.38	
Durbin-Watson	1.74	
No. of observations	90	

Table 3 shows the multiple linear regression analysis to model the relationship between the impedance index (H^2^/R) and predictor variables (age, gender, Morita Dehydration Score, oedema presence, urea:creatinine ratio and the Burge-4 score).

### Hydration assessment and BIVA

**Fig 3:**
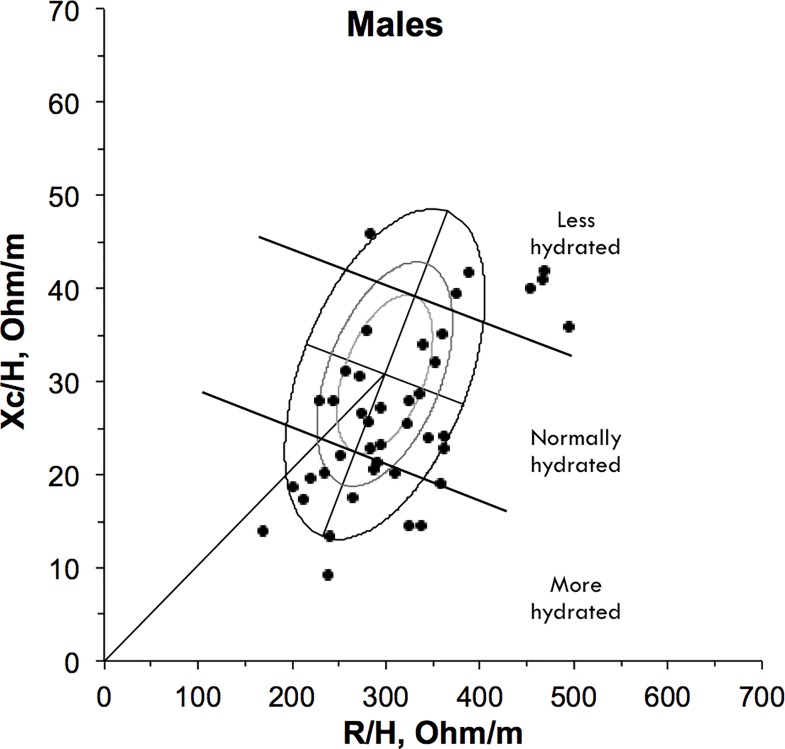
Vector positions for males on the RXc point graph (N = 42). Values for male participants are illustrated by circles on the 50%, 75%, and 95% bioimpedance tolerance ellipses of the reference population.

**Fig 4:**
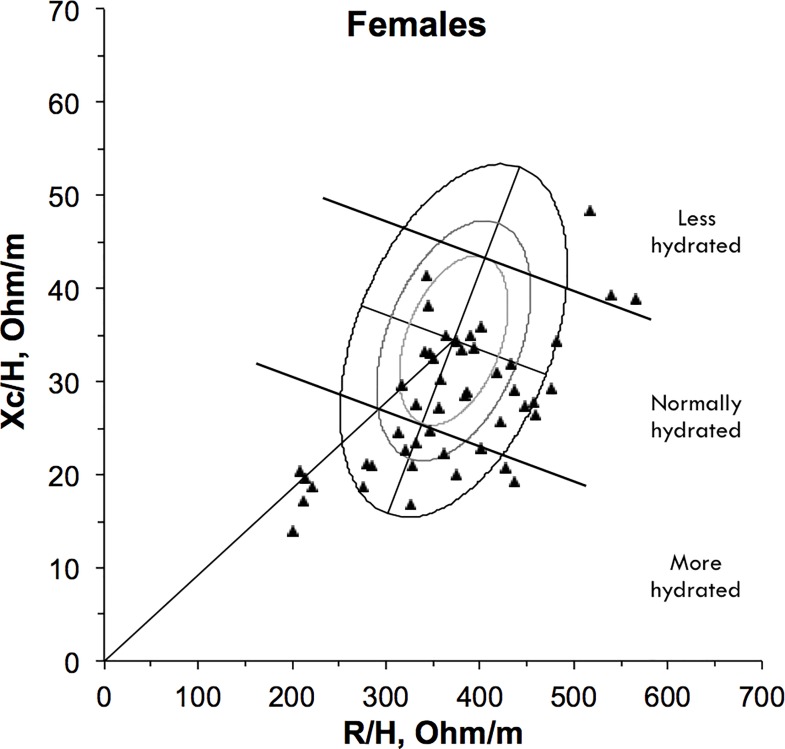
Vector positions for females on the RXc point graph (N = 48). Values for female participants are illustrated by triangles on the 50%, 75%, and 95% bioimpedance tolerance ellipses of the reference population.

Hydration status was normal in 43 (47.8%), ‘more-hydrated’ in 37 (41.1%) and ‘less hydrated’ in 10 (11.1%) (Figs 3 and 4; Table 4). We simplified the hydration status classifications to compare ‘less-hydrated’ participants (n = 10, 11.1%) to those ‘not less hydrated’ (n = 80, 88.9%). This comparison demonstrated that oral fluid intake was statistically significantly lower in ‘less hydrated’ participants compared to those ‘not less hydrated’ (p = 0.04) (Table 5). Additionally, axilla dryness scored significantly higher in those ‘less hydrated’ compared to those ‘not less hydrated’ (p = 0.02). No other statistically significant differences were detected for the current sample. The analysis demonstrated non-significant differences between the groups, with ‘less hydrated’ individuals reporting higher values (when compared to the ‘not less hydrated’ group) for symptoms (the Burge-4 sub-scores), the Morita Dehydration Score, the ur:cr ratio, urinary osmolality and ECOG.

**Table 4:** Classification of hydration as a three-item scale according to the RXC graph scale.

Hydration status	Male	Female	Total (%)
Normal	18	25	43 (47.8)
‘Less hydrated’	7	3	10 (11.1)
‘More hydrated’	17	20	37 (41.1)
**Total**	42	48	90

Table 4 shows the hydration status of participants according to the position of individual vectors on the RXc graph scale.

**Table 5:** Comparison between ‘less hydrated’ and ‘not less hydrated’ groups.

	‘Less hydrated’ (n = 10)		‘Not less hydrated’ (n = 80)			
**Variable**	**Mean**	**SD**	**Mean**	**SD**	**T test**	**p**
Burge-4 score (mm)	257.60	91.76	217.63	95.47	1.25	0.21
*Thirst*	72.70	22.97	54.04	29.67	1.92	0.06
*Dry mouth*	70.60	25.80	58.69	31.07	1.16	0.25
*Unpleasant taste*	52.20	37.51	41.11	33.71	0.97	0.34
*Fatigue*	62.10	23.73	63.79	30.92	-0.17	0.87
Ur:Cr ratio (mmol/mmol)	137.15	101.33	91.62	42.20	1.41	0.19
Na (mmol/L) (*n = 89*)	134.10	4.77	136.38	4.17	-1.60	0.11
eGFR (mL/min/1.73m^2^)	71.00	24.68	72.24	18.09	-0.20	0.85
AdjCa (mmol/L) (n = 61)	2.26	0.13	2.32	0.25	-0.81	0.42
Serum osmalilty (mosm/kg)	281.75	18.46	286.68	9.38	-0.95	0.35
Albumin (g/L)	29.70	3.95	32.36	6.25	-1.31	0.19
Urine osmolality (mosm/kg) (n = 61)	540.50	177.03	505.39	212.25	0.31	0.76
H^2^/R (m^2^/Ohm)	39.57	9.28	53.08	15.40	-2.71	0.008
**Mann U Whitney test variable**	**Mean**	**Rank**	**Mean**	**Rank**	**Z**	**P**
ECOG	52.90	529.00	44.58	3566.00	326	0.32
Oral intake (mLs)	31.25	312.5	47.28	3782.50	257.5	0.04
Morita Dehydration Score	58.60	586.00	43.86	3509.00	269	0.08
*Mucous*	52.45	524.50	44.63	3570.50	330.5	0.34
*Axilla dryness*	61.00	610.00	43.56	3485.00	245	0.02
*Sunken eyes*	44.90	449.00	45.58	3646.00	394	0.92

Table 5 shows a comparison between the ‘less hydrated’ and the ‘not less hydrated’ groups according to symptoms (Burge-4 score), biochemical measures, performance status (ECOG), oral fluid intake and physical symptoms (Morita Dehydration Score).

### Survival analysis

**Fig 5:**
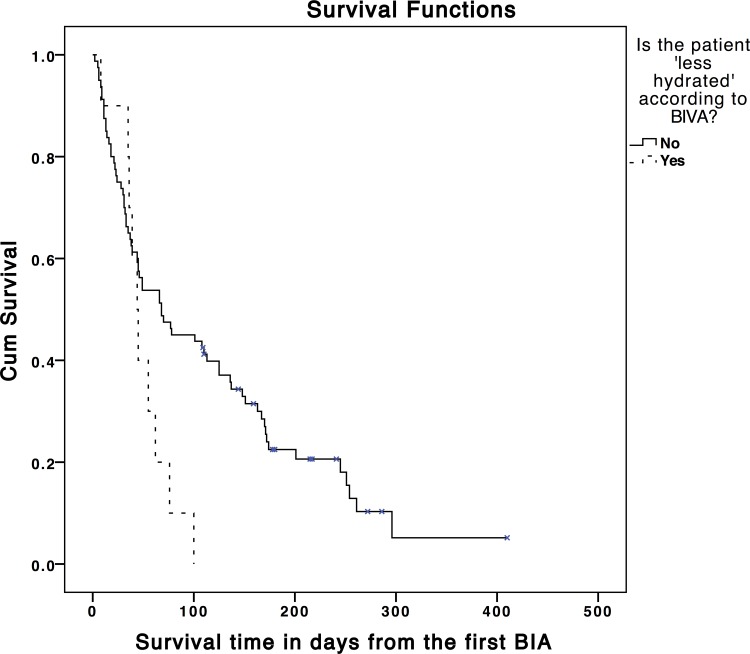
Kaplan-Meier graph showing survival time in days according to the ‘less hydrated’ classification (χ^2^ = 4.08, P = 0.04). Tick marks indicate censoring of data.

**Fig 6:**
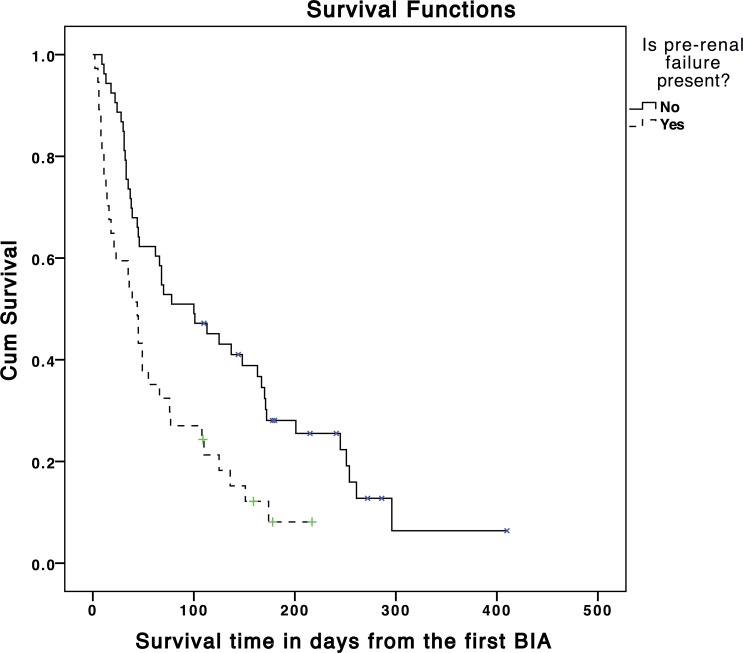
Kaplan-Meier graph showing survival time in days according to the presence or absence of pre-renal failure (χ^2^ = 8.99, P = 0.003). Tick marks indicate censoring of data.

Seventy-six (84.4%) participants died before the end of the follow-up period. Median survival for the sample was 62 days (Table 6). Median survival was shortest in ‘less hydrated’ participants (44 days) and longest in those ‘more hydrated’ (70 days). Survival was statistically significantly shorter when ‘less hydrated’ participants were compared to those ‘not less hydrated’ (44 days vs. 68 days, hazard ratio = 2.01 [95% Confidence Interval (CI) = 1.00, 4.02]; p = 0.049) (Fig 5). Participants with pre-renal failure had shorter survival when compared to those without pre-renal failure (44 days vs. 100 days, hazard ratio = 2.03 [95%CI = 1.26, 3.26]; p = 0.003) (Fig 6).

**Table 6:** Univariate survival analysis of participants according to hydration status and pre-renal failure classifications.

Subgroup	Median survival in days	Hazard ratio (95% CI)	p
Overall	62.0		
*Hydration classification according to three BIVA classifications*			
Normal	68.0	1.00 (ref)	0.09
‘Less hydrated’	44.0	2.01 (1.00, 4.02)	0.11
‘More hydrated’	70.0	0.72 (0.45, 1.15)	0.34
*Hydration classification according to two BIVA classifications*			
‘Not less hydrated’	68.0	1.00 (ref)	
‘Less hydrated’	44.0	2.01 (1.00, 4.02)	0.049
*Pre-renal failure present*?			
No	100.0	1.00 (ref)	
Yes	44.0	2.03 (1.26, 3.26)	0.003

Table 6 shows the survival analysis data for participants according to hydration status and pre-renal failure classifications.

The H^2^/R and the ur:cr ratio remained significant predictors of survival following statistical adjustment (cox regression) for age, sex, baseline ECOG performance status and cancer type (Table 7). Cancer type also significantly influenced survival p = 0.02. The hazard ratio for death according to the H^2^/R was 0.98 [95%CI = 0.96, 1.00] (p = 0.04); this means each unit m^2^/Ohm increase of the H^2^/R was associated with reduced probability of death by a factor of 1.02 (i.e. 1/hazard ratio). The hazard ratio for death according to the ur:cr ratio was 1.01 [95%CI = 1.00, 1.02], p = 0.001; this means that each unit increase (mmol:mmol) of the ur:cr ratio was associated with an increased probability of death by a factor of 0.99.

**Table 7:** Multivariate cox regression analysis for death according to age, performance status, cancer type, the ur:cr and the H^2^/R.

Variable	Hazard Ratio (95% CI)	p
Age (years)	0.99 (0.97, 1.02)	0.52
Female	0.84 (0.47, 1.51)	0.56
ECOG 1		0.06
ECOG (2 vs. 1)	1.69 (0.72, 3.96)	0.23
ECOG (3 vs. 1)	2.82 (1.22, 6.54)	0.02
ECOG (4 vs. 1)	3.07 (1.17, 8.07)	0.02
Cancer type (GI)		0.02
Cancer type (Gyne vs. GI)	0.40 (0.13, 1.24)	0.11
Cancer type (Lung vs. GI)	1.35 (0.67, 2.75)	0.41
Cancer type (Misc vs. GI)	0.41 (0.20, 0.83)	0.01
Cancer type (GU vs GI)	0.42 (0.18, 1.00)	0.05
Ur:Cr (mmol/mmol)	1.01 (1.00, 1.02)	0.001
H^2^/R (m^2^/Ohm)	0.98 (0.96, 1.00)	0.04

Table 7 shows the cox regression analysis for death (hazard ratio) according to age, performance status (ECOG), cancer type, the ur:cr and H^2^/R. Abbreviations: GI = gastrointestinal; Gyne = gynecological; Misc = miscellaneous; GU = genitourinary.

## Discussion

### Main findings and new knowledge

This is the first study to use BIVA to evaluate hydration and its relationship with symptoms and survival in advanced cancer patients. The findings demonstrate that hydration (as measured by H^2^/R and BIVA) in advanced cancer was significantly associated with physical signs (mucous membrane moisture, axilla dryness, sunken eyes, oedema), symptoms (dry mouth, thirst, unpleasant taste, fatigue) and oral fluid intake. Survival was statistically significantly shorter in ‘less-hydrated’ individuals and those with pre-renal failure.

### Comparison with previous work

Our data demonstrates that the Morita Dehydration Score was associated with hydration status, which supports previous work using this tool to assess physical signs of hydration.[32] In this study, women had a lower H^2^/R compared to men, which suggests comparatively lower hydration volume. This finding is consistent with previous work[6, 51] and human physiology,[6, 51, 58, 59] as women normally have more body fat than men and therefore comparatively have less body water in proportion to their weight.[58, 59] Oedematous participants had higher H^2^/R compared to non-oedematous participants, which suggests they had an increased hydration volume. Although these findings are consistent with the literature it is not possible to determine intracellular or extracellular volumes without the use of regression equations; however, these equations have methodological limitations in advanced cancer.[24, 25, 60–62]

Our study demonstrates how higher Burge-4 scores were associated with lower H^2^/R (which suggests comparatively lower TBW volume). Consequently, our data supports previous work concerning the assessment of dehydration symptoms in advanced cancer[36, 40] and non-cancer populations.[63–67] Our analysis demonstrates that lower oral fluid intake was associated with ‘less hydrated’ patients. However, we are unable to determine whether this reduction in oral intake contributed to the participant’s hydration volume, or if it was the result of their clinical condition.

Previous estimates of cancer dehydration prevalence are generally based on biochemical criteria with the prevalence reported to be 60–75%.[68, 69] In this study, only 11.1% of participants were ‘less-hydrated’ and 41.1% were ‘more hydrated’. Comparatively, the prevalence of pre-renal failure in this study was 41.1%, which is consistent with previous work.[54] Our findings demonstrated that (in this sample) individuals were more likely to be ‘more hydrated’ as opposed to ‘less hydrated’. This may suggest that biochemical definitions of dehydration lack sensitivity in people with advanced cancer. Furthermore, the regression analysis did not detect a statistically significant association between the ur:cr ratio and the H^2^/R variables (Table 3). This data supports previous work that demonstrates how biochemical measures poorly correlate with symptoms (dry mouth, thirst, fatigue and unpleasant taste) in advanced cancer.[2, 3, 33, 53, 69, 70] This provides evidence that biochemical measures lack sensitivity to predict hydration-related symptoms in advanced cancer.

### Survival

Our data demonstrates that pre-renal failure was associated with shorter survival in patients with advanced cancer, which is consistent with previous research.[71] Similarly, the Prognosis in Palliative care Study (PiPS) reported how an elevated urea measurement was a predictor of shorter survival in patients with advanced cancer.[72] We are unable to determine the exact reason for the association of shorter survival with lower H^2^/R and BIVA hydration status. However, it is possible that ‘less hydrated’ individuals were more likely to have a clinical picture that was associated with shorter survival (e.g. cachexia).

### Limitations

This study describes a small, predominantly Caucasian, specialist palliative care population in the last two months of life. Only ten participants were ‘less hydrated’, which meant that the two-group analysis was statistically limited. Consequently, the ability to extrapolate the results of this analysis to other population groups is limited. This study was observational and therefore is unable to determine causation of the studied variables. This analysis involved many different cancers at various stages of illness. Survival was significantly influenced by cancer type, meaning some participants experienced shorter survival on account of their diagnosis. Consequently, the exact influence of hydration on survival is difficult to determine. Furthermore, it is possible that pre-existing differences (related to the different cancers) may have caused variation in the body composition of participants. Although the H^2^/R is the single best predictor of TBW we are unable estimate fluid volume without the use of regression equations (which improve the accuracy of TBW measurement but are methodologically limited in cancer). Additionally, the BIVA method is unable to distinguish between fluid compartments or define intracellular and extracellular fluid volumes, which limits our ability to study these differences in greater detail.

### What is the significance of the findings of this analysis?

This analysis provides evidence that hydration status is related to physical signs (mucous membrane moisture, axilla dryness, sunken eyes, oedema), symptoms (dry mouth, thirst, unpleasant taste, fatigue), oral fluid intake and survival in an advanced cancer population. These variables can potentially be used to develop criteria for the assessment of hydration status, for the purposes of research and clinical practice. Our data reports no statistically significant association between biochemical measures and hydration status. Furthermore, we report that (in this sample) a greater number of participants were ‘more-hydrated’ compared to those ‘less-hydrated’. Consequently, we recommend that healthcare professionals should carefully assess hydration status in their patients; it may be possible for individuals to be at risk of fluid overload even though the biochemical results indicate pre-renal failure. However, we are unable to provide recommendations for the use (or non use) of CAH as this was beyond the scope of this research study.

### Future opportunities and research possibilities

Future studies can build on this work and use BIVA to study hydration status according to specific cancers, stratified by performance status, stage of illness, ethnicity and gender. The non-invasive properties of BIVA provide the potential to conduct longitudinal assessments to study hydration and symptoms over time [28] (e.g. the dying phase), in order to determine the clinical utility of CAH. Further studies can examine the potential use of bioimpedance for prognostication.

## Conclusions

In advanced cancer, hydration status (classified by BIA/BIVA) relates to clinically measurable signs and symptoms. Hydration status and pre-renal failure were independent predictors of survival. Further studies can establish the utility of BIVA as a standardised hydration assessment tool and explore its potential research application, in order to inform the clinical management of fluid balance in patients with advanced cancer.

### Ethics

Written consent was obtained from all study participants; this included consent to report individual patient data in publication. Participant consent was recorded in a research recruitment log. All participants were provided with a copy of their consent forms. This study and the consent procedure were approved by the North Wales Research Ethics Committee–West (Local research ethics committee approval number = 12/WA/0200). The study was sponsored by the University of Liverpool. The researchers were independent from funders and sponsors. The sponsors and funder did not have access to the data or a role in the data analysis, results interpretation or writing the manuscript.

## Supporting Information

S1 Dataset Minimal anonymized dataset for the study findings. (SAV)

## Abbreviations

BIA: bioelectrical impedance analysis
BIVA: bioelectrical impedance vector analysis
CAH: clinical assisted hydration
ECOG: Eastern Cooperative Oncology Group performance status
H: height
M: mean
R: resistance
RXc: resistance reactance
SNRI: Serotonin and norepinephrine reuptake inhibitors
SSRI: Selective serotonin re-uptake inhibitors
Xc: reactance
TBW: total body water
